# Additivity of digestible energy and nutrient concentrations in hatchery byproducts fed to nursery pigs

**DOI:** 10.5713/ab.21.0124

**Published:** 2021-06-24

**Authors:** Jung Yeol Sung, Sang Yun Ji, Beob Gyun Kim

**Affiliations:** 1 Department of Animal Science and Technology, Konkuk University, Seoul 05029, Korea; 2 Animal Nutritional Physiology Team, National Institute of Animal Science, Rural Development Administration, Wanju 55363, Korea

**Keywords:** Additivity, Hatchery Byproduct, Nutrient Utilization, Swine

## Abstract

**Objective:**

The objective was to test additivity of digestible energy and nutrient concentrations in the hatchery byproduct mixture fed to nursery pigs.

**Methods:**

In the previous studies, energy, phosphorus, calcium, and amino acid digestibility of infertile eggs, unhatched eggs, culled chicks, and a mixture of 3 hatchery byproduct ingredients was determined in nursery pigs (initial body weight = 9.4 to 14.2 kg). An additivity test was conducted using these determined values.

**Results:**

No difference was observed between determined and predicted metabolizable energy values in the mixture (3,998 and 3,990 kcal/kg as-is basis, respectively). Measured standardized total tract digestible phosphorus in the mixture was less than the predicted value (4.5 vs 5.3 g/kg as-is basis, respectively; p<0.05). Measured standardized total tract digestible calcium in the mixture was greater compared with the predicted value (40.0 vs 31.7 g/kg as-is basis, respectively; p<0.05). Measured standardized ileal digestible tryptophan in the mixture was greater than the predicted value (3.7 vs 3.1 g/kg as-is basis, respectively; p<0.05) whereas other amino acid values were additive.

**Conclusion:**

Energy and most of amino acid concentrations in hatchery byproducts are additive in the mixture fed to nursery pigs.

## INTRODUCTION

Additivity is the fundamental assumption when formulating diets using multiple ingredients. If a digestible energy (DE) value or a nutrient concentration in a mixed diet is equal to the proportional sum of DE values or nutrients from each ingredient, DE or nutrient concentration in the diet is regarded additive [1]. In layers hatchery facilities, infertile eggs, unhatched eggs, and culled chicks are regularly disposed of because only female chicks are economically valuable. These wastes from hatchery facilities are known as hatchery byproducts [2] and are regarded as alternative protein sources in nursery pig diets. A hatchery byproduct mixture could replace animal protein sources without compromising growth performance of nursery pigs [3]. For these reasons, the use of hatchery byproducts in swine diets would be both economically and environmentally beneficial. Generally, hatchery byproducts are pooled and then discarded together, but the ratio of each byproduct is not always constant.

An accurate nutritional evaluation of a feed ingredient is necessary for precise feed formulations [4,5]. To evaluate nutritional values of feed ingredients precisely, *in vivo* experiments with animals are required. However, it is nearly impossible to conduct *in vivo* experiments to determine nutritional value of hatchery byproduct mixtures whenever the ratio in the mixture changes. If the ratio of each component in the pooled hatchery byproduct mixture in layer hatchery facilities is known or estimated and if the energy and nutrient values are additive, available energy and nutrient concentrations in the mixture would be calculated as the proportional sum of energy and nutrients from each ingredient resulting in a precise feed formulation [1,6]. Therefore, the objective of the present study was to test the additivity of digestible energy, phosphorus (P), calcium (Ca), and amino acid (AA) concentrations in a hatchery byproduct mixture fed to nursery pigs based on previous metabolism studies [4,7].

## MATERIALS AND METHODS

All protocols used in the animal studies were approved by the Animal Care and Use Committee of Konkuk University (approval number: KU17049, KU18072, and KU18145). Four digestibility experiments were conducted in environmentally controlled rooms at Konkuk University.

### Data collection

Digestible energy, metabolizable energy (ME), apparent total tract digestible (ATTD) P, standardized total tract digestible (STTD) P, ATTD Ca, STTD Ca, apparent ileal digestible (AID) AA, and standardized ileal digestible (SID) AA in infertile eggs, unhatched eggs, culled chicks, and a mixture of 3 ingredients consisted of 200 g/kg infertile eggs, 200 g/kg unhatched eggs, and 600 g/kg culled chicks (Table 1) fed to nursery pigs (initial body weight = 9.4 to 14.2 kg) were determined in our previous experiments [4,7]. The experimental diets for DE and ME measurement had constant corn to whey powder ratio to enable the calculation of DE and ME in the test ingredients using difference procedure (Table 2). The diets for P, Ca, and AA digestibility determination were formulated to contain a test ingredient as the sole source of P, Ca, or AA (Tables 3, 4, and 5), and thus, P, Ca, and AA digestibility values in an experimental diet represented the digestibility values in the ingredient. For energy, P, and Ca studies, the marker-to-marker method was used to collect feces totally, whereas the index method was used in AA study. Antibiotics were not included in the experimental diets.

### Calculations and statistical analyses

Digestible energy in infertile eggs, unhatched eggs, and culled chicks measured in the previous study [4] were used for calculating the predicted DE (kcal/kg) in the mixture according to the following equation modified from Kong and Adeola [8]:


$$\text{Predicted }{\text{DE}}_{\text{Mixture}}=({\text{DE}}_{\text{Infertile eggs}}+{\text{DE}}_{\text{Unhatched eggs}}+3\times {\text{DE}}_{\text{Culled chicks}})/5$$

where predicted DE_Mixture_ is predicted DE in the mixture and DE_Infertile eggs_, DE_Unhatched eggs_, and DE_Culled chicks_ are measured DE values in infertile eggs, unhatched eggs, and culled chicks, respectively. Predicted values for ME (kcal/kg) and digestible P, Ca, and AA (g/kg) in the mixture were calculated as same as DE using the reported values [4,7].

For the comparison between measured and predicted values, a t-test was used and an alpha level of 0.05 was used to determine significance [9].

## RESULTS

In the previous studies, 2 batches of hatchery byproducts were obtained and dried separately [4,7]. The first batch was used in energy and P digestibility experiments and the second batch was employed for Ca and AA digestibility experiments. Analyzed compositions of each hatchery byproduct of 2 batches were comparable (Table 1). Energy concentration and nutrient utilization of each hatchery byproduct ingredient and the mixture are provided in Table 6.

No difference between measured and predicted values for DE and ME in the hatchery byproduct mixture of infertile eggs, unhatched eggs, and culled chicks was observed (Table 7). While measured ATTD P and STTD P in the mixture were less than predicted values (p<0.05), measured ATTD Ca and STTD Ca were greater than predicted values (p<0.05). No difference was observed between measured and predicted AID and SID AA in the mixture except for tryptophan (Table 8).

## DISCUSSION

Information on additivity of energy concentrations in animal protein sources for non-ruminants is very limited. In ruminants, energy concentrations may not be additive if a mixed diet is composed of forages and readily available carbohydrates due to changes in pH value, volatile fatty acid production, and microbial activity in the rumen which significantly affect energy metabolism [10]. In contrast to ruminants, the DE and ME values in feed ingredients for pigs are known to be additive in the mixed diets containing feed ingredients including corn, soybean meal, camelina cake, and wheat bran [11,12]. The additivity assumption on energy concentrations in swine diets may be violated if the proportion of a high-fiber ingredient in a mixed diet is very high likely due to negative effects of dietary fiber on energy digestibility of other ingredients. If a high-fiber ingredient in a mixed diet impedes energy utilization of other ingredients in the mixed diets, an actual energy concentration in the mixed diet would be less than the value calculated assuming additivity. In the present work, energy concentrations were additive in the hatchery byproduct mixture as hatchery byproducts are animal protein sources containing very little fiber.

The hatchery byproduct mixture used by Sung et al [4,7] contained infertile eggs, unhatched eggs, and culled chicks. The classification of each hatchery byproduct ingredient is based on the hatchery process. Infertile eggs are the eggs identified as infertile on day 8 post-fertilization. Fertile eggs are incubated until day 21 post-fertilization. Unhatched eggs are the eggs where embryonic development ceases or a chick does not break eggshells until day 21 post-fertilization. Culled chicks are weak chicks or male chicks that cannot lay eggs.

The STTD P or Ca and SID AA have been reported to be more additive compared with ATTD and AID nutrients in the mixture [13–15]. The reason for the non-additivity of apparent digestible nutrients in the mixture is an underestimation of ingredient nutrient digestibility particularly when a nutrient concentration is low mainly due to basal endogenous losses of nutrients [1,6]. In contrast to the ATTD and AID nutrients, STTD and SID nutrients are independent of basal endogenous losses of nutrients because these values are calculated by correcting apparent digestible nutrients for basal endogenous losses [16–19]. For this reason, NRC [20] suggested that diet formulations and requirements should be expressed on STTD P and SID AA.

In the present work, predicted ATTD and STTD P in the mixture were greater than the measured values, indicating that digestible P concentrations in the hatchery byproducts are not additive in the mixture. The non-additivity of digestible P in the mixture may be partially due to different Ca to P ratios among the experimental diets. Phosphorus digestibility in pigs was reported to linearly decrease as the dietary Ca concentration increased from 7 to 23 g/kg when dietary P concentration was below the requirement [21]. The lower P digestibility with high Ca to P ratio in diets is likely due to the Ca-P complex formed by increased dietary Ca, which eventually impedes P from being absorbed. The contribution of P provided by culled chicks accounts for the largest proportion of total P (approximately 73%) in the mixture and Ca to P ratio in the mixture diet is more than twice of that in culled chick diet (4.4 vs 2.0). The low P digestibility in the mixture diet appears to be mainly attributed to the large Ca to P ratio. The P digestibility measured in the culled chick diet perhaps was not reflected in the mixed diet. In other studies, however, standardized or true total tract P digestibility values in mixed diets containing various cereal grains and oilseed meals were additive [13,22,23]. The Ca to P ratios in the experimental diets used in those studies ranged from 0.6 to 1.5, indicating that a non-excessive Ca to P ratio in diets is important for the additivity of digestible P in the mixture.

In contrast to P, predicted ATTD and STTD Ca were less than measured values. The reason for the non-additivity of digestible Ca in the mixture may be also attributed to different Ca to P ratios among experimental diets. Calcium intake exceeding the requirement has been suggested to lower intestinal absorption of Ca [24,25]. As P concentrations in experimental diets were relatively constant in the present work, dietary Ca intake was dependent on Ca to P ratio in diets. Calcium to P ratio in the mixture diet was less than that in infertile egg diet and unhatched egg diet (1.8 vs 2.6 and 2.7). Compared with the daily Ca requirement suggested by NRC [20] for 11- to 25-kg pigs (6.3 g/d), dietary Ca intake of pigs fed to infertile egg diet and unhatched egg diet (8.1 and 7.8 g/d) exceeded the requirement whereas Ca intake of the mixture diet was 5.8 g/d which was less than the requirement. The reason for greater measured digestible Ca in the mixture may be due to that the contribution of Ca supplied by infertile eggs and unhatched eggs in the mixture was over 80% and that the downregulation of intestinal Ca absorption of infertile eggs and unhatched eggs may have been alleviated in the mixture diet. However, Stein et al [21] reported that Ca digestibility was not compromised when the Ca to P ratio in the diet and daily Ca intake of 23-kg pigs were up to 2.3 and 9.7 g/d which are comparable to the values in the previous study [7]. The reason for the inconsistent results is unclear. Zhang and Adeola [14] reported that true total tract digestibility of Ca was additive in the Ca-P balanced mixed diets containing limestone and dicalcium phosphate in 20-kg pigs. In their experiment [14], the amount of dietary Ca was below the daily Ca requirement (2.3 to 3.6 g/d vs 6.3 g/d) and Ca to P ratios were relatively constant (0.9 to 1.0).

No difference between measured and predicted AID and SID AA except for tryptophan in the mixture was observed. Each hatchery byproduct was the sole source of the respective experimental diet when determining AID and SID AA. The AID AA were not additive in the literature [1,15]. The major reason for the non-additivity was low AA concentrations in the ingredients. In the present work, however, each hatchery byproduct contains relatively high concentrations of AA, and thus, AID values in the mixture could have been relatively precisely predicted from each ingredient. Standardized ileal digestible AA are widely accepted to be additive in mixed diets [6,15,23]. The reason for non-additivity of SID tryptophan in the mixture remains unclear. The low tryptophan concentration in experimental diets possibly resulted in large errors in the measurement of tryptophan digestibility [6]. However, this speculation is not supported by the previous studies where tryptophan concentration was also the lowest among AA in experimental diets [6,15,21].

## CONCLUSION

Taken together, digestible energy and most AAs in hatchery byproducts were additive in the mixture fed to nursery pigs. However, digestible tryptophan, phosphorus, and calcium concentrations in the hatchery byproduct mixture deviated from the values calculated based on digestible nutrients in the ingredients.

## Figures and Tables

**Table 1:** Analyzed composition of hatchery byproducts, as-is basis^1)^

Item	Infertile eggs		Unhatched eggs		Culled chicks		Mixture^2)^	
			
	Batch 1	Batch 2	Batch 1	Batch 2	Batch 1	Batch 2	Batch 1	Batch 2
Dry matter (g/kg)	911	969	973	984	983	973	965	972
Gross energy (kcal/kg)	4,206	4,630	4,313	4,296	5,884	5,897	5,192	5,342
Crude protein (g/kg)	322	396	377	400	675	663	574	555
Ether extract (g/kg)	229	260	216	237	208	225	214	236
Ash (g/kg)	319	313	356	357	87	85	168	188
Calcium (g/kg)	120	116	129	127	18	18	61	62
Phosphorus (g/kg)	5.6	5.2	6.5	5.5	11.1	9.9	9.4	8.3
Indispensable amino acids (g/kg)								
Arginine	-	21.2	-	21.0	-	40.4	-	30.8
Histidine	-	7.6	-	7.3	-	12.6	-	10.1
Isoleucine	-	16.8	-	15.1	-	23.3	-	19.5
Leucine	-	30.9	-	28.4	-	47.4	-	38.6
Lysine	-	22.7	-	20.9	-	34.4	-	28.3
Methionine	-	10.5	-	9.7	-	11.1	-	10.5
Phenylalanine	-	18.4	-	17.0	-	25.9	-	21.8
Threonine	-	17.3	-	16.2	-	25.9	-	21.3
Tryptophan	-	4.6	-	3.7	-	5.8	-	5.4
Valine	-	20.7	-	19.0	-	29.0	-	24.3
Dispensable amino acids (g/kg)								
Alanine	-	20.2	-	19.7	-	34.1	-	27.1
Aspartic acid	-	35.7	-	32.9	-	50.6	-	42.3
Cysteine	-	7.5	-	7.4	-	13.7	-	11.3
Glutamic acid	-	46.0	-	44.5	-	75.9	-	60.5
Glycine	-	13.5	-	16.2	-	44.2	-	30.1
Proline	-	15.0	-	15.7	-	32.8	-	24.1
Serine	-	25.3	-	22.9	-	35.0	-	29.4
Tyrosine	-	13.2	-	12.1	-	20.2	-	16.5

1) Analyzed composition of batches 1 and 2 were adapted from Sung et al [4,7], respectively. The batch 1 was used for the determination of energy and phosphorus digestibility. The batch 2 was used for calcium and amino acid digestibility experiments.

2) The mixture contained 200 g/kg dried infertile eggs, 200 g/kg dried unhatched eggs, and 600 g/kg dried culled chicks.

**Table 2:** Ingredient and chemical compositions of the experimental diets used in energy digestibility experiment, as-fed basis^1)^

Item	Diet				

	Basal	Infertile egg	Unhatched egg	Culled chick	Mixture^2)^
Ingredient (g/kg)					
Ground corn	867.0	660.1	660.1	656.7	662.8
Whey powder	100.0	76.1	76.1	75.7	76.4
Infertile eggs	-	250.0	-	-	-
Unhatched eggs	-	-	250.0	-	-
Culled chicks	-	-	-	250.0	-
Mixture	-	-	-	-	250.0
Others^3)^	33.0	13.8	13.8	17.6	10.8
Analyzed composition					
Dry matter (g/kg)	904	909	921	920	919
Gross energy (kcal/kg)	3,850	3,971	4,006	4,369	4,274
Crude protein (g/kg)	95	157	164	237	207

1) Adapted from Sung et al [4,7].

2) The mixture contained 200 g/kg dried infertile eggs, 200 g/kg dried unhatched eggs, and 600 g/kg dried culled chicks.

3) Others were limestone, monosodium phosphate, sodium chloride, vitamin premix, and mineral premix.

**Table 3:** Ingredient and chemical compositions of the experimental diets used in phosphorus digestibility experiment, as-fed basis^1)^

Item	Diet				

	Infertile egg	Unhatched egg	Culled chick	Mixture^2)^	P-free
Ingredient (g/kg)					
Corn starch	452.5	462.5	535.6	502.5	520.2
Infertile eggs	250.0	-	-	-	-
Unhatched eggs	-	250.0	-	-	-
Culled chicks	-	-	250.0	-	-
Mixture	-	-	-	250.0	-
Sucrose	200.0	200.0	200.0	200.0	200.0
Gelatin	90.0	80.0	-	40.0	150.0
Others^3)^	7.5	7.5	14.4	7.5	129.8
Analyzed composition					
Dry matter (g/kg)	923	935	941	933	927
Gross energy (kcal/kg)	3,984	3,943	4,241	4,127	3,998
Crude protein (g/kg)	175	176	171	180	186
Calcium (g/kg)	28.9	28.7	6.7	12.3	5.7
Phosphorus (g/kg)	1.9	2.0	3.4	2.8	0.1

1) Adapted from Sung et al [4,7].

2) The mixture contained 200 g/kg dried infertile eggs, 200 g/kg dried unhatched eggs, and 600 g/kg dried culled chicks.

3) Others were cellulose, soybean oil, crystalline amino acids, limestone, potassium carbonate, magnesium oxide, sodium chloride, vitamin premix, and mineral premix.

**Table 4:** Ingredient and chemical compositions of the experimental diets used in calcium digestibility experiment, as-fed basis^1)^

Item	Diet				

	Infertile egg	Unhatched egg	Culled chick	Mixture^2)^	Ca-free
Ingredient (g/kg)					
Ground corn	764.7	769.8	733.9	778.0	828.9
Potato protein concentrate	115.0	110.0	-	50.0	150.0
Infertile eggs	100.0	-	-	-	-
Unhatched eggs	-	100.0	-	-	-
Culled chicks	-	-	250.0	-	-
Mixture	-	-	-	150.0	-
Others^3)^	20.3	20.2	16.1	22.0	21.1
Analyzed composition					
Dry matter (g/kg)	882	882	892	883	871
Gross energy (kcal/kg)	4,019	4,000	4,327	4,068	4,027
Crude protein (g/kg)	203	199	216	182	196
Calcium (g/kg)	12.4	11.8	4.8	9.0	0.9
Phosphorus (g/kg)	4.8	4.4	4.6	4.9	4.6

1) Adapted from Sung et al [4,7].

2) The mixture contained 200 g/kg dried infertile eggs, 200 g/kg dried unhatched eggs, and 600 g/kg dried culled chicks.

3) Others were monosodium phosphate, sodium chloride, vitamin premix, and mineral premix.

**Table 5:** Ingredient and chemical compositions of the experimental diets used in amino acid digestibility experiment, as-fed basis^1)^

Item	Diet				

	Infertile egg	Unhatched egg	Culled chick	Mixture^2)^	N-free
Ingredient (g/kg)					
Cornstarch	479.3	480.1	479.9	482.5	680.0
Sucrose	200.0	200.0	200.0	200.0	200.0
Infertile eggs	300.0	-	-	-	-
Unhatched eggs	-	300.0	-	-	-
Culled chicks	-	-	300.0	-	-
Mixture	-	-	-	300.0	-
Others^3)^	20.7	19.9	20.1	17.5	120.0
Analyzed composition					
Dry matter (g/kg)	936	940	936	936	925
Gross energy (kcal/kg)	3,920	3,824	4,302	4,111	3,681
Crude protein (g/kg)	119	119	200	160	3
Lysine (g/kg)	7.0	6.8	10.1	8.3	0.1
Methionine (g/kg)	3.2	2.9	3.2	3.2	0.1
Threonine (g/kg)	5.4	5.3	7.7	6.4	0.1
Tryptophan (g/kg)	1.5	1.5	1.4	1.5	0.0

1) Adapted from Sung et al [4,7].

2) The mixture contained 200 g/kg dried infertile eggs, 200 g/kg dried unhatched eggs, and 600 g/kg dried culled chicks.

3) Others were soybean oil, cellulose, limestone, monosodium phosphate, potassium carbonate, magnesium oxide, sodium chloride, vitamin-mineral premix, and chromium oxide.

**Table 6:** Energy concentrations (as-is basis) and coefficient of nutrient digestibility of phosphorus (P), calcium (Ca), and amino acids (AA) in the hatchery byproducts^1)^,^2)^

Item^3)^	Infertile egg	Unhatched egg	Culled chick	Mixture^4)^	SEM	p-value
Gross energy (kcal/kg)	4,206	4,313	5,884	5,192	-	-
Digestible energy (kcal/kg)	2,759^d^	3,735^c^	4,840^a^	4,224^b^	116	<0.001
Metabolizable energy (kcal/kg)	2,645^c^	3,625^b^	4,560^a^	3,998^b^	123	<0.001
CATTD of P	0.640^a^	0.447^b^	0.438^b^	0.355^b^	0.042	<0.001
CSTTD of P	0.817^a^	0.616^b^	0.539^b^	0.474^b^	0.042	<0.001
CATTD of Ca	0.485^b^	0.383^c^	0.633^a^	0.580^a^	0.033	<0.001
CSTTD of Ca	0.533^c^	0.434^d^	0.756^a^	0.645^b^	0.033	<0.001
CSID of crude protein	0.750^a^	0.713^ab^	0.638^b^	0.682^ab^	0.027	0.023
CSID of indispensable AA						
Arginine	0.815^a^	0.791^ab^	0.717^b^	0.738^ab^	0.024	0.009
Histidine	0.783^a^	0.750^ab^	0.683^b^	0.715^ab^	0.027	0.027
Isoleucine	0.820^a^	0.779^ab^	0.675^c^	0.718^bc^	0.028	<0.001
Leucine	0.842^a^	0.804^ab^	0.706^c^	0.749^bc^	0.027	0.001
Lysine	0.810	0.793	0.777	0.795	0.026	0.740
Methionine	0.836	0.810	0.809	0.834	0.024	0.606
Phenylalanine	0.824^a^	0.782^ab^	0.670^c^	0.716^bc^	0.027	<0.001
Threonine	0.788^a^	0.750^ab^	0.652^b^	0.700^ab^	0.030	0.010
Tryptophan	0.833^a^	0.774^ab^	0.513^c^	0.694^b^	0.030	<0.001
Valine	0.820^a^	0.780^ab^	0.662^c^	0.709^bc^	0.027	<0.001
CSID of dispensable AA						
Alanine	0.797	0.779	0.726	0.750	0.028	0.163
Aspartic acid	0.719^a^	0.665^ab^	0.593^b^	0.626^b^	0.025	0.006
Cysteine	0.723^a^	0.623^ab^	0.381^c^	0.525^b^	0.032	<0.001
Glutamic acid	0.810^a^	0.782^ab^	0.701^b^	0.742^ab^	0.026	0.017
Glycine	0.645	0.656	0.586	0.598	0.047	0.465
Proline	0.195	0.317	0.363	0.363	0.185	0.701
Serine	0.741^a^	0.714^a^	0.563^b^	0.620^b^	0.025	<0.001
Tyrosine	0.829^a^	0.779^ab^	0.635^c^	0.694^bc^	0.031	<0.001

SEM, standard error of the means; CATTD, coefficient of apparent total tract digestibility; CSTTD, coefficient of standardized total tract digestibility; CSID, coefficient of standardized ileal digestibility.

1) Adapted from Sung et al [4,7].

2) Each least squares mean represents 6 to 8 observations.

3) Basal endogenous losses (g/kg dry matter intake) were determined from pigs fed semi-purified diets: phosphorus, 0.36; calcium, 0.66; crude protein, 21.52; arginine, 0.69; histidine, 0.27; isoleucine, 0.44; leucine, 0.75; lysine, 0.73; methionine, 0.14; phenylalanine, 0.47; threonine, 0.92; tryptophan, 0.18; valine, 0.67; alanine, 0.86; aspartic acid, 1.17; cysteine, 0.30; glutamic acid, 1.58; glycine, 1.56; proline, 3.11; serine, 0.91; tyrosine, 0.40.

4) The mixture contained 200 g/kg dried infertile eggs, 200 g/kg dried unhatched eggs, and 600 g/kg dried culled chicks.

a–d Least squares means within a row without a common superscript differ (p<0.05).

**Table 7:** Measured and predicted values for energy, phosphorus (P), and calcium (Ca) concentrations in the hatchery byproduct mixture, as-is basis

Item	Measured	Predicted	Standard error^1)^	p-value
DE (kcal/kg)	4,224	4,203	199	0.890
ME (kcal/kg)	3,998	3,990	219	0.959
ATTD P (g/kg)	3.3	4.2	0.2	0.003
STTD P (g/kg)	4.5	5.3	0.2	0.004
ATTD Ca (g/kg)	36.0	27.9	1.9	0.004
STTD Ca (g/kg)	40.0	31.7	1.9	0.004

DE, digestible energy; ME, metabolizable energy; ATTD, apparent total tract digestible; STTD, standardized total tract digestible.

1) Standard error of the difference between measured and predicted values.

**Table 8:** Measured and predicted values for digestible crude protein and amino acid (AA) concentrations (g/kg) in the hatchery byproduct mixture, as-is basis

Item	Apparent ileal digestible				Standardized ileal digestible			
	
	Measured	Predicted	SE^1)^	p-value	Measured	Predicted	SE	p-value
Crude protein	308.7	303.2	12.5	0.469	378.7	370.4	12.5	0.356
Indispensable AA								
Arginine	20.5	21.9	0.7	0.113	22.8	24.2	0.7	0.125
Histidine	6.3	6.6	0.3	0.557	7.2	7.4	0.3	0.621
Isoleucine	12.5	13.2	0.6	0.583	14.0	14.6	0.6	0.634
Leucine	26.5	27.5	1.1	0.667	28.9	29.9	1.1	0.705
Lysine	20.1	20.8	0.8	0.700	22.5	23.0	0.8	0.752
Methionine	8.3	8.3	0.3	0.565	8.8	8.7	0.3	0.603
Phenylalanine	14.1	14.6	0.7	0.671	15.6	16.1	0.7	0.712
Threonine	12.0	12.5	0.7	0.731	14.9	15.3	0.7	0.805
Tryptophan	3.1	2.5	0.2	0.004	3.7	3.1	0.2	0.004
Valine	15.0	15.8	0.7	0.487	17.2	17.9	0.7	0.558
Dispensable AA								
Alanine	17.6	18.5	0.7	0.404	20.3	21.1	0.7	0.474
Aspartic acid	22.8	23.9	1.1	0.474	26.5	27.5	1.1	0.521
Cysteine	5.0	4.2	0.4	0.067	5.9	5.1	0.4	0.063
Glutamic acid	40.0	41.5	1.6	0.548	44.9	46.3	1.6	0.587
Glycine	13.1	14.7	0.9	0.169	18.0	19.4	0.9	0.221
Proline	−0.5	−0.3	4.0	0.930	8.7	8.7	4.0	0.967
Serine	15.4	16.1	0.8	0.533	18.2	18.8	0.8	0.585
Tyrosine	10.1	10.4	0.6	0.848	11.5	11.8	0.6	0.896

SE, standard error.

1) Standard error of the difference between measured and predicted values.
